# Mutation in Mg-Protoporphyrin IX Monomethyl Ester (Oxidative) Cyclase Gene *ZmCRD1* Causes Chlorophyll-Deficiency in Maize

**DOI:** 10.3389/fpls.2022.912215

**Published:** 2022-07-07

**Authors:** Yingjie Xue, Haixiao Dong, Hongru Huang, Shipeng Li, Xiaohui Shan, He Li, Hongkui Liu, Dong Xia, Shengzhong Su, Yaping Yuan

**Affiliations:** Jilin Engineering Research Center for Crop Biotechnology Breeding, College of Plant Science, Jilin University, Changchun, China

**Keywords:** maize, chlorophyll-deficiency, positional mapping, *ZmCRD1*, chlorophyll biosynthesis, photosynthesis

## Abstract

Chlorophyll molecules are non-covalently associated with chlorophyll-binding proteins to harvest light and perform charge separation vital for energy conservation during photosynthetic electron transfer in photosynthesis for photosynthetic organisms. The present study characterized a *pale-green leaf* (*pgl*) maize mutant controlled by a single recessive gene causing chlorophyll reduction throughout the whole life cycle. Through positional mapping and complementation allelic test, *Zm00001d008230* (*ZmCRD1*) with two missense mutations (p.A44T and p.T326M) was identified as the causal gene encoding magnesium-protoporphyrin IX monomethyl ester cyclase (MgPEC). Phylogenetic analysis of ZmCRD1 within and among species revealed that the p.T326M mutation was more likely to be causal. Subcellular localization showed that ZmCRD1 was targeted to chloroplasts. The *pgl* mutant showed a malformed chloroplast morphology and reduced number of starch grains in bundle sheath cells. The *ZmCRD1* gene was mainly expressed in WT and mutant leaves, but the expression was reduced in the mutant. Most of the genes involved in chlorophyll biosynthesis, chlorophyll degradation, chloroplast development and photosynthesis were down-regulated in *pgl*. The photosynthetic capacity was limited along with developmental retardation and production reduction in *pgl*. These results confirmed the crucial role of *ZmCRD1* in chlorophyll biosynthesis, chloroplast development and photosynthesis in maize.

## Introduction

Chlorophylls (Chls) are one of the most abundant tetrapyrrole molecules. Chls not only harvest light, but are also involved in energy transfer and are essential for charge separation within photosystems (Stuart et al., 2020). More than 17 enzymes are involved in the chlorophyll biosynthesis pathway, which is composed of 5′-aminolevulinic acid (ALA) formation, protoporphyrin IX formation, chlorophyll *a* (Chl *a*) formation and chlorophyll a/b cycle (Czarnecki and Grimm, 2012). Researchers have identified two different ALA synthesis pathways, including the C4 pathway and C5 pathway (involved in the chlorophyll branch), in organisms (Panek and O’Brian, 2002). Glutamyl-tRNA synthetase, glutamyl-tRNA reductase and glutamate 1-semialdehyde aminotransferase are involved in the C5 pathway to form ALA using glutamate and tRNA*^Glu^* as substrates. Then, protoporphyrin IX is synthesized by six enzymes (ALA dehydratase, hydroxymethylbilane synthase, uroporphyrinogen III synthase, uroporphyrinogen III decarboxylase, coproporphyrinogen III oxidase and protoporphyrinogen IX oxidase) using ALA as a precursor. Mg-chelatase and MgPEC play an important role in the chlorophyllide *a* (Chlide *a*) formation pathway. Mg^2+^ is inserted into protoporphyrin IX forming Mg-protoporphyrin IX through a process mediated by Mg-chelatase, which is converted to Mg-protoporphyrin IX monomethyl ester (Mg-protoME) by Mg-protoporphyrin IX methyltransferase. The fifth ring of divinyl protochlorophyllide (DV Pchlide) is formed by MgPEC using Mg-protoME as a substrate. Chlide *a* is synthesized by divinyl protochlorophyllide reductase and protochlorophyllide oxidoreductase from DV Pchlide (Masuda and Fujita, 2008; Brzezowski et al.,, 2015). In the chlorophyll a/b cycle, Chl *a* and chlorophyll *b* (Chl *b*) are interconverted to each other (Tanaka and Tanaka, 2011; Voitsekhovskaja and Tyutereva, 2015). Chlide *a* serves as a precursor for Chl *a*, which process is catalyzed by chlorophyll synthase and then converted to Chl *b* by chlorophyll *a* oxygenase. Chl *b* can be reconverted to Chl *a* by Chl *b* reductase and 7-hydroxymethyl Chl *a* reductase (Czarnecki and Grimm, 2012). Chl *a* and Chl *b* mainly exist in higher plants and algae, but Cyanophyta lacks Chl *b*. In higher plants, Chl *a* exists in core complexes and light harvesting complexes (LHC), while Chl *b* only exists in LHC (Green and Durnford, 1996; Jia, 2021). Most chlorophyll molecules are non-covalently bound to photosynthetic proteins on the thylakoid membrane to form chlorophyll-protein antennas that capture solar energy, and a few Chl *a* (special pairs of P680 and P700) molecules can excite an electron used for photosynthetic electron transport in the reaction centers of PS II and PS I (Jansson, 2004; Stefano et al., 2014; Voitsekhovskaja and Tyutereva, 2015). When plants suffer from environmental stresses damaging photosystems, especially PS II, Chls are turned over in green leaves and are involved in the repair of damaged PS II. Chl *a* is mainly affected in Chls turnover. Chl *a* and Phetin *a* perform Chls turnover and *de novo* biosynthesis to promptly repair PS II in response to changes of environment (Feierabend and Dehne, 1996; Beisel et al., 2010; Lin et al., 2016; Jia, 2021). In addition, Chl *b* plays a crucial role in the correct assembly of antenna complexes in thylakoids. The stability of antenna complexes depends on Chl *b*, and the availability of Chl *b* affects the number of minor antenna proteins (Voitsekhovskaja and Tyutereva, 2015). Therefore, Chl *a* and Chl *b* perform common or specific functions in plants.

Leaf color mutants are ideal materials to explore molecular regulatory mechanisms in chlorophyll biosynthesis, chloroplast development and photosynthesis. Previous studies have identified a series of leaf color mutants in various species, including maize (Huang et al., 2009; Xing et al., 2014), rice (Chen et al., 2018; Li et al., 2018), wheat (Wu et al., 2018), barley (Wang R. et al., 2017; Xu et al., 2019), *Arabidopsis* (Chai et al., 2005; Asakura et al., 2008), sorghum (Kawahigashi et al., 2016), and soybean (Campbell et al., 2014). Most leaf color mutants are loss-of-function mutations that exhibit lower chlorophyll contents, abnormal chloroplast morphology and reduced photosynthetic capacity, and a few mutants are gain-of-function mutations with advantages that include improvements in photosynthesis and crop yields under certain conditions (Gu J. et al., 2017; Gu J. F. et al., 2017; Kirst et al., 2017).

Most of the enzymes involved in chlorophyll biosynthesis have been identified and are clearly known, but components of MgPEC are poorly understood in maize. MgPEC is first studied in cucumber and extracted to transform MgPME to Mg-2,4-divinyl pheoporphyrin A(5) *in vitro* (Chereskin and Castelfranco, 1982). MgPEC utilizes two different catalytic mechanisms in photosynthetic organisms in the presence of various concentrations of oxygen: a usual aerobic mechanism under high oxygen concentrations and an anaerobic mechanism under low or oxygen-free conditions that correspond to oxygen-dependent MgPEC and oxygen-independent MgPEC, respectively. Oxygen-independent MgPEC is encoded by a single gene *BchE* in bacteriochlorophyll biosynthesis (Chereskin and Castelfranco, 1982; Wong and Castelfranco, 1984; Beale, 1999; Ouchane et al., 2004). Oxygen-dependent MgPEC is composed of at least four components including AcsF/XanL, Ycf54, membrane-associated Viridis-k and a soluble component. Its activity is associated with ferredoxin and ferredoxin-NADPH oxidoreductase in barley (Rzeznicka et al., 2005; Bollivar et al., 2014; Stuart et al., 2020). *CHL27* is a homologous gene of *Chlamydomonas Crd1* that encodes a subunit of aerobic cyclase in *Arabidopsis*. Antisense *Arabidopsis* mutant lines exhibit various degrees of chlorophyll-deficient phenotypes following reduced accumulation of chlorophyll-binding proteins (Tottey et al., 2003; Bang et al., 2008). Rice pale-green leaf mutants *m167* and *ysl1* display yellow-green leaves during the whole growth period, and the chlorophyll content and photosynthetic capacity are lower in mutants than wild type plants due to the mutation of *OsCRD1* (Wang X. X. et al., 2017; Li et al., 2019). EMS-mutagenized cucumber mutants have pale green leaves and fruits controlled by a recessive allele of *CsYcf54* that encodes a Ycf54-like protein required for MgPEC (Lun et al., 2016). *Epipremnum aureum* mutants exhibit a chlorophyll-deficient phenotype controlled by the nuclear gene *EaZIP* that is a homologous gene of *Arabidopsis CHL27* and tobacco *NTZIP*, and *AtCHL27* can restore its green phenotype and chloroplast development (Hung et al., 2010, 2021).

The present study identified a mutated gene, *ZmCRD1*, encoding MgPEC that is involved in chlorophyll biosynthesis in a novel chlorophyll-deficient maize mutant. Mutation of *ZmCRD1* caused abnormal chloroplast morphology, perturbed photosynthesis and reduced production. The results presented here showed that *ZmCRD1* is crucial for chlorophyll biosynthesis and will facilitate further research on chlorophyll biosynthesis and photosynthesis in maize.

## Materials and Methods

### Plant Materials and Construction of the Mapping Population

The EMS mutagenesis was performed in the maize inbred line B73 to generate a series of mutants. A *pale-green leaf* (*pgl*) mutant was identified from this population and used in this study. The *pgl* mutant was crossed with B73 and then self-crossed to construct the F_2_ genetic segregation population to identify the candidate gene controlling leaf color.

### Measurement of Photosynthetic Pigment Contents and Chlorophyll Fluorescence Parameters

The third leaves of B73 and *pgl* were collected at the third-leaf stage. Leaf samples were immersed in 30 ml of leach liquor composed of acetone and ethanol (volume ratio was 2:1) at 25°C for 24 h in the dark. The absorbance (OD) was measured using ultraviolet-visible spectrophotometer (WFZ UV-2800AH, UNICO, Shanghai, China) at 663, 645, and 470 nm. Photosynthetic pigment contents, Chl *a*, Chl *b*, Chls and carotenoid (Car), were calculated using the following formulas (Arnon, 1949):

(1) Chl *a* (mg/g) = (12.72 × OD_663_–2.69 × OD_645_) × V × N/W
(2) Chl *b* (mg/g) = (22.88 × OD_645_–4.68 × OD_663_) × V × N/W
(3) Chls (mg/g) = Chl *a* + Chl *b*
(4) Car (mg/g) = [OD470 × (V/W)–3.27 × Chl *a*–104 × Chl *b*]/198

V, N, and W represent the volume of the leach liquor, dilution times and fresh weight, respectively.

The chlorophyll fluorescence kinetics parameters were measured using a multifunctional plant measurement instrument MultispeQ system (MultispeQ V2, United States) at the middle of canopy leaves or ear leaves in the sunny forenoon from 8:00 to 11:30 am in Changchun, China. The actinic light used sunlight on the same day that light intensity was measured via the PAR sensor of MultispeQ instrument (V8 stage: 112.75–542.42 μmol photons m^–2^ s^–1^; V10 stage: 95.05–517.73 μmol photons m^–2^ s^–1^; V15 stage: 106.23–184.36 μmol photons m^–2^ s^–1^; V16 stage: 250.07–655.44 μmol photons m^–2^ s^–1^; R2 stage: 135.58–351.28 μmol photons m^–2^ s^–1^; R3 stage: 76.21–152.94 μmol photons m^–2^ s^–1^; R4 stage: 32.80–97.68 μmol photons m^–2^ s^–1^; R5 stage: 162.57–422.42 μmol photons m^–2^ s^–1^; and R6 stage: 173.07–544.82 μmol photons m^–2^ s^–1^). The parameters were calculated by the instrument using the following formulas:

(1) Relative chlorophyll content = k × {log_10_[(Abs_940*nm*_ × ref.Abs_650*nm*_)/(Abs_650*nm*_ × ref.Abs_940*nm*_)]} (Maas and Dunlap, 1989; Raymond and Daughtry, 2014)
(2) *F*_*v*_/*F*_*m*_ = (*F*_*m*_ − *F*_*o*_)/*F*_*m*_ (Genty et al., 1989)
(3) Phi2 = (*F*′_*m*_ − *F*_*s*_)/*F*′_*m*_ (Genty et al., 1989)
(4) qL = (*F*′_*m*_ − *F*_*s*_)/(*F*′_*m*_ − *F*′_*o*_) × (*F*′_*o*_/*F*_*s*_) (Kramer et al., 2004)
(5) PhiNPQ = 1 − (*F*′_*m*_ − *F*_*s*_)/*F*′_*m*_ − *F*_*s*_/*F*_*m*_ (Kuhlgert et al., 2016)
(6) gH^+^ = 1/τ (Kanazawa and Kramer, 2002)
(7) The fraction of active PSI is expressed as the ratio of *P*_*M*_/*P*_0_ (Kanazawa et al., 2017)

Detail explanations of these parameters are provided in Supplementary Table 1.

### RNA-Seq and Bulked Segregant Analysis

A wild-type pool (WP) of 200 normal leaf plants and a mutant-type pool (MP) of 200 pale-green leaf plants were collected from the B73/*pgl* F_2_ population. These two pools were subjected to RNA-seq. RNA extraction, library preparation and sequencing were performed by Biomarker (Beijing, China) using the Illumina Navo 6000 platform.

Bioinformatics analysis was performed using a common approach. The high-quality reads (clean reads) were aligned and mapped to the B73_RefGen_v4 reference genome^1^ (Jiao et al., 2017) using HISAT2^2^ (Kim et al., 2015). Variant callings were performed using GATK (McKenna et al., 2010). SnpEff was used for variant effect annotation (Cingolani et al., 2012). SNP-index values were used for gene mapping (Abe et al., 2012). The SNP-index at a position was calculated for each pool using the following formula: SNP-index = (number of reads supporting the mutated allele)/(total number of reads covering this position). The difference between two pools (ΔSNP-index = SNP-index*^MP^* – SNP-index*^WP^*) was calculated and analyzed.

### Genotype Using the High Resolution Melting Method

An additional 885 F_2_ individuals with pale-green leaves were subjected to genomic DNA extraction separately using the modified CTAB method (Rogers and Bendich, 1989). Polymorphic SNPs were selected and each individual was genotyped using HRM. PCR and resolution melting were performed using Illumina Eco Quantitative Real-Time PCR System and 2 × Super EvaGreen Master Mix (US EVERBRIGHT INC., Suzhou, China). The following thermal cycling conditions used: 95°C for 2 min, followed by 45 cycles of 95°C for 5 s and 60°C for 30 s, finally, one cycle of 95°C for 15 s, 60°C for 60 s, with an increase of 0.3°C/cycle to 95°C and 95°C for 15 s.

### Complementation Allelic Test

A maize mutant of *ZmCRD1*, *Zmcrd1*, was obtained from the maize mutator insertional library (ChinaMu). *Zmcrd1* contains a mutator transposon inserted in the first exon of *ZmCRD1*. The genotype of *Zmcrd1* was identified by PCR using *Zmcrd1* DNA as a template (Supplementary Table 2, primers #5 and #6). The heterozygous *Zmcrd1* and wild-type plants were crossed with B73, *pgl* and B73/*pgl* F_1_ progenies. The phenotypes of their crossing progenies were identified and the segregation ratio was counted. Seedlings were grown to the third-leaf stage in greenhouse and then subjected to phenotyping.

### Protein Conservation Analysis

The MgPEC protein CRD1 was searched in the protein database of NCBI^3^ among different species and 168 proteins were selected for multiple sequence alignment and phylogenetic analysis. Multiple sequence alignment was performed using Clustal Omega (Fábio et al., 2019) and the results were visualized using Mview^4^. The phylogenetic tree was visualized using MAGE7 (Sudhir et al., 2016) and iTOL (Ivica and Peer, 2019). ZmCRD1 was blast against the protein database in MaizeGDB^5^ within species, and 37 proteins were selected for multiple sequence alignment. The transcriptome data of MgPEC genes spanning various tissues and stages in maize (Walley et al., 2016) were obtained from the public database qTeller^6^.

### Subcellular Localization Analysis

The subcellular localization of target genes was performed by transient expression in mesophyll protoplasts of *Arabidopsis* as previously reported (Yoo et al., 2007). The longer precursors containing the coding sequence (CDS) of *ZmCRDs* (*ZmCRD1* or *ZmCRD2*) were cloned using the B73 cDNA as a template (Supplementary Table 2, primers #1 and #3). Precursor of *ZmCRD1*_*mut*_ (ZmCRD1 with p.A44T and p.T326M mutations) was cloned using the *pgl* cDNA as a template (Supplementary Table 2, primer #1). The full CDS of *ZmCRD1*, *ZmCRD1*_*mut*_ and *ZmCRD2* were cloned using their longer precursors as templates (Supplementary Table 2, primers #2, #2, and #4) and then cloned into the pSATN1-GW vector using the Gateway Cloning System (Invitrogen) to express fusion proteins with GFP-tag at the C-terminus. *Arabidopsis* plants (Col-0 ecotype) were grown under a relatively short photoperiod (12 h light at 23°C/12 h dark at 21°C) with 50% relative humidity for 4 weeks. The recombinant vectors were transformed into mesophyll protoplasts using the PEG/Ca^2+^ method. The transformed protoplasts were incubated at room temperature in the dark for 12–16 h and then observed using an inverted fluorescence microscope (Nikon, Japan).

### Transmission Electron Microscopy Analysis

Fresh leaves of B73 and *pgl* were collected at the third-leaf stage and grown in greenhouse. The leaves were cut into fragments of approximately 1 cm^2^ and then fixed with a 2.5% (v/v) glutaraldehyde solution overnight. After staining with uranyl acetate, the samples were dehydrated with ethanol solutions of different concentrations and cut into thin sections. Finally, the ultrastructure of chloroplasts was observed with a transmission electron microscope (JEM1200, JEOL, Japan). Three biological replications were performed for each sample.

### RNA Extraction and Quantitative Real-Time PCR Analysis

Total RNA was extracted from all samples using an ultrapure RNA kit (CWBIO, Beijing, China). First-strand cDNA was generated using PrimeScript RT reagent Kit with gDNA Eraser (Perfect Real Time, TaKaRa, Beijing, China). PCR was performed using Illumina Eco Quantitative Real-Time PCR System and 2 × SYBR Green qPCR Master Mix (Bimake, Shanghai, China). The following thermal cycling conditions were used: 95°C for 30 s, followed by 40 cycles of 95°C for 5 s and 60°C for 34 s, finally, one cycle of 95°C for 15 s, 60°C for 60 s, with an increase of 0.3°C/cycle to 95°C and 95°C for 15 s. The relative expression levels of genes were calculated using the 2^–Δ^ ^Δ^ *^Ct^* method and *ZmTUB4* (NCBI accession: NM_001111987.1) was used as an internal control for normalization.

## Results

### *pgl* Is a Chlorophyll-Deficient Maize Mutant

**FIGURE 1:**
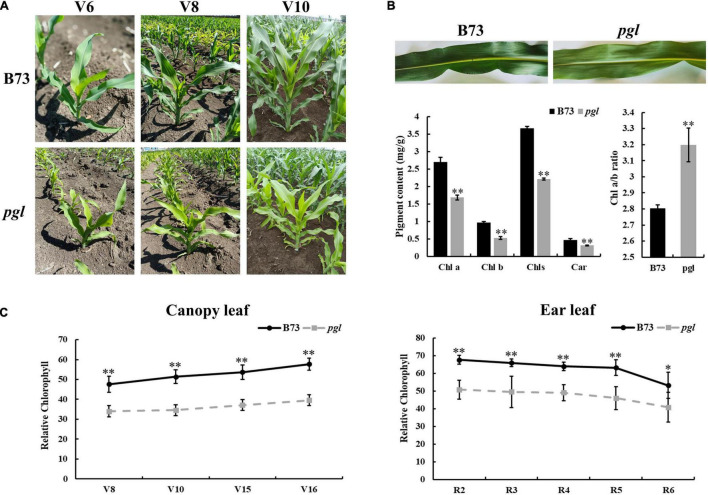
Phenotypic characterization of the *pgl* mutant. **(A)** B73 and *pgl* were observed at different stages. V6, sixth leaf stage; V8, eighth leaf stage; V10, tenth leaf stage. **(B)** Pigment contents of the third leaf were measured in B73 and *pgl* using absorption photometry. Seedlings were grown to the third-leaf stage in greenhouse. Data are presented as the means ± SD (*n* = 3). **(C)** Relative chlorophyll content was measured and compared at different stages between B73 and *pgl*. V15, fifteenth leaf stage; V16, sixteenth leaf stage; R2, blister stage; R3, milk stage; R4, dough stage; R5, dent stage; R6, physiological maturity stage. Data are presented as the means ± SD (*n* = 9). Asterisks represent significant differences between B73 and *pgl* detected by independent sample T test (*P < 0.05; ^**^P < 0.01).

A novel *pale-green leaf* (*pgl*) mutant was identified from EMS induced maize mutant library of inbred line B73. The *pgl* mutant showed pale green leaves throughout its lifespan (Figure 1A). The contents of chlorophyll pigments in *pgl* were significantly reduced compared to B73. The accumulation levels of Chls, Chl *a*, Chl *b* and Car decreased to 60.49, 62.59, 55.21, and 62.75% of B73, respectively, and the Chl a/b ratio increased (Figure 1B), which showed that *PGL* locus mutation had more effects on Chl *b* synthesis and affected ratio of chlorophyll composition in *pgl*. The relative chlorophyll content showed an increasing trend at the vegetative growth stage, but decreased at the reproductive stage (Figure 1C). The *pgl* mutants were crossed with wild-type B73 plants to generate F_2_ segregation populations. All F_1_ offspring exhibited a normal green leaf phenotype, and the F_2_ population showed a segregation ratio of 3:1 (green plant: pale green plant) (Supplementary Table 3). These results indicated that a single recessive gene caused the chlorophyll-deficient phenotype in *pgl*.

### *ZmCRD1* as a Candidate Gene in *pgl*

**FIGURE 2:**
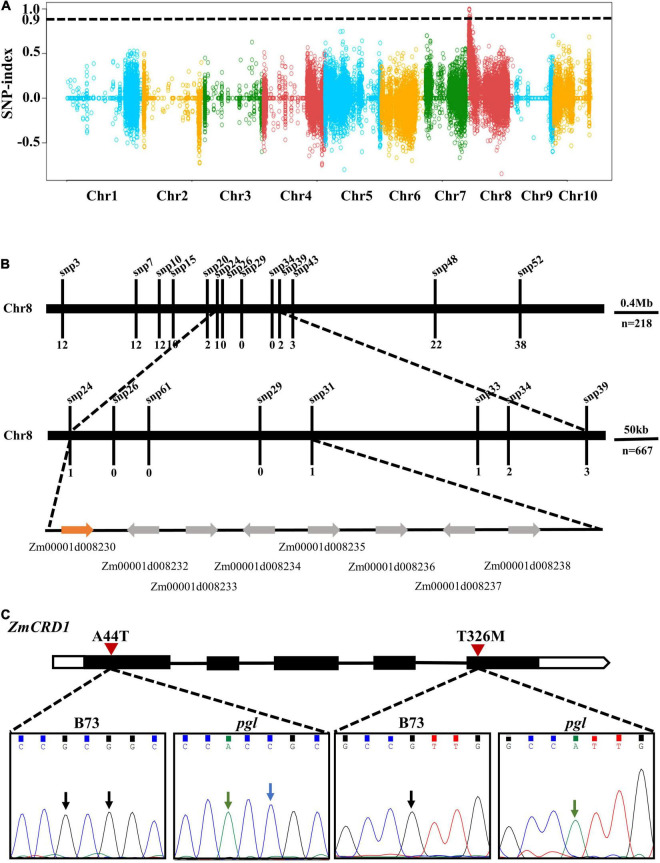
Positional mapping of the *PGL* locus. **(A)** The *PGL* locus was initially mapped on chromosome 8 based on the BSA strategy and RNA-seq. The black dotted line is the threshold value (0.9). Dots of different colors belong to different chromosomes. **(B)** The *PGL* locus was finely mapped to a narrow interval of approximately 274 kb using HRM. **(C)** Gene model and mutation site analysis of the candidate gene *ZmCRD1*. The black rectangles represent exons, lines represent introns, and the white regions represent UTRs. Arrows point to missense mutation sites between B73 and *pgl*.

To map the causal gene, *pgl* was crossed with wild-type B73 plants. We performed RNA-seq on a wild-type pool (WP) and a mutant-type pool (MP) of F_2_ progenies (Supplementary Table 4). Bulked segregant analysis (BSA) indicated one obvious peak on chromosome 8 (from 0.56 to 4.78 Mb) and the region was 4.22 Mb containing multiple genes and variants (Figure 2A). To further narrow the interval of the *PGL* locus, sixteen polymorphic SNPs were selected within this region (Supplementary Table 5). A total of 885 (218 + 667) *pgl*-like F_2_ progenies were genotyped using the HRM method to screen recombinant plants. One recombinant plant was identified using markers snp24 and snp31, while the number of recombinant plants identified using the markers snp26, snp29, and snp61 was zero. Therefore, the *PGL* locus was finally mapped between markers snp24 and snp31 representing a 274 kb region (chr8: 1983989-2258462) (Figure 2B).

There were eight protein-coding genes in this region, and all of them were cloned and sequenced. Two non-synonymous mutations were detected at the first and fifth exons of *Zm00001d008230*. These mutations were alanine (A) to threonine (T) change at the 44th position (p.A44T) and T to methionine (M) change (p.T326M) at the 326th position of the protein (Figure 2C). The *Zm00001d008230* gene was annotated to encode a catalytic subunit of magnesium-protoporphyrin IX monomethyl ester cyclase and named as *ZmCRD1*.

### Validation of *ZmCRD1* Using a Complementation Allelic Test

**FIGURE 3:**
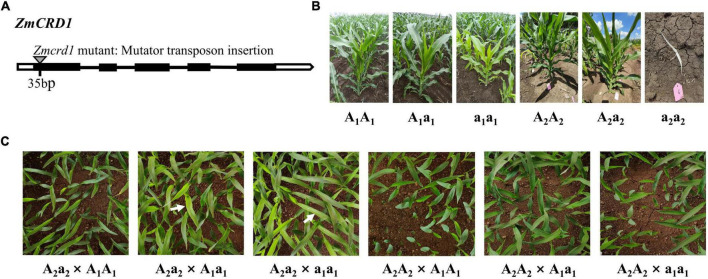
The functional complementation assay between the *pgl* mutant and *Zmcrd1* mutant. **(A)** The structure model of mutator insertion in the *ZmCRD1* gene. **(B)** Phenotypic identification of mutational individuals of different genotypes used for the complementation allelic test. **(C)** Genetic analysis of *pgl* and *Zmcrd1* associated F_2_ populations. Seedlings were grown to the third-leaf stage in greenhouse and then subjected to phenotyping. The white arrows point to the individuals with mutated phenotype.

We obtained another maize mutant of *ZmCRD1* (named *Zmcrd1*) from the maize mutator insertional library (ChinaMu) to further confirm that *ZmCRD1* gene was the causal gene in *pgl*. A mutator transposon was inserted in the first exon of *ZmCRD1* in the *Zmcrd1* mutant (Figure 3A). Heterozygous *Zmcrd1* mutants (A_2_a_2_) exhibited a green leaf phenotype and homozygous mutants (a_2_a_2_) were albino lethal (Figure 3B). The heterozygous *Zmcrd1* and wild-type plants (A_2_A_2_) were crossed with B73 (A_1_A_1_), *pgl* (a_1_a_1_) and B73/*pgl* F_1_ (A_1_a_1_) for allelic test (Figure 3B). The progenies of A_2_a_2_ × a_1_a_1_ showed a segregation ratio of 1:1 (green plant: pale green plant). The progenies of A_2_a_2_ × A_1_a_1_ showed a segregation ratio of 3:1 (green plant: pale green plant). While progenies of other cross combinations exhibited a green leaf phenotype (Figure 3C and Supplementary Table 6). All these results confirmed that the *PGL* gene was an allele of *ZmCRD1* and mutations of *ZmCRD1* led to chlorophyll deficiency.

### p.T326M Is More Likely to Be the Causal Mutation

**FIGURE 4:**
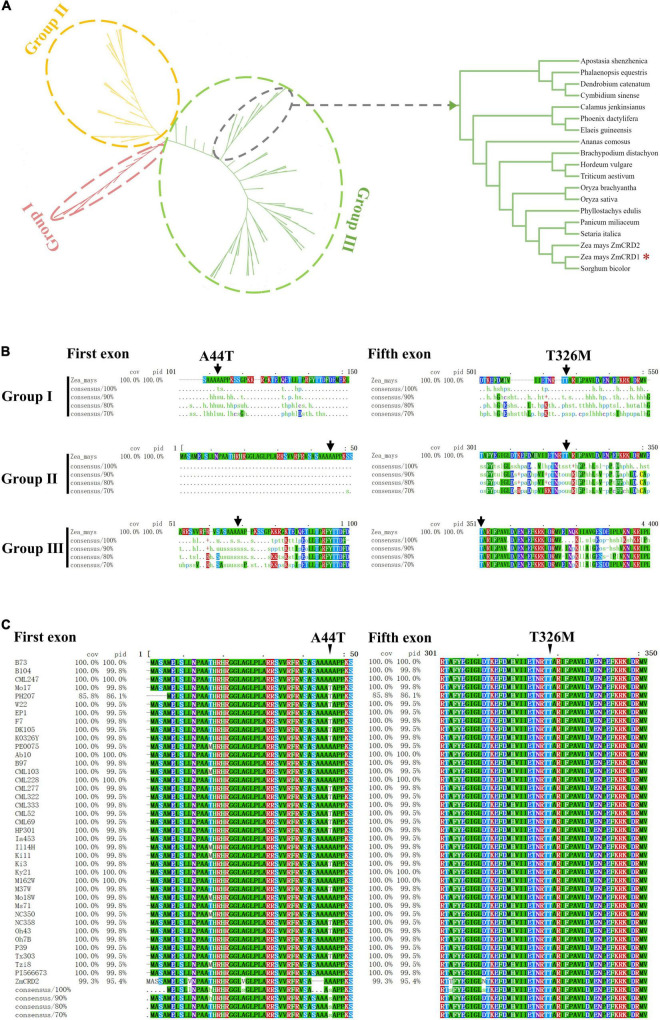
The conservation and phylogenetic analysis of CRD1. **(A)** Phylogenetic analysis of ZmCRD1 and its 168 homologous proteins. The right image is an enlarged view of 18 CRD proteins containing ZmCRD1. The detailed annotated information of 18 CRD proteins is available in Supplementary Table 7. **(B)** Multiple sequence alignment of ZmCRD1 and its homologous proteins within each subgroup. The arrows point to the p.A44 and p.T326 sites of ZmCRD1. **(C)** Multiple sequence alignment of ZmCRD1 in 37 maize inbred lines. The arrows point to the p.A44 and p.T326 sites of ZmCRD1.

Two missense mutations, p.A44T and p.T326M, were detected in ZmCRD1, but it remained unknown which one was the causal or they both took effects. Phylogenetic analysis was performed on ZmCRD1 and its homologous proteins from 168 species belonging to 17 prokaryotes and 151 eukaryotes. Phylogenetic analysis indicated that ZmCRD1 and its homologous proteins were clearly divided into three classifications (I, II, and III). Group I included all bacteria and archaebacteria belonging to prokaryotes. Group II was primarily composed of algae. Group III consisted of higher plants and a tiny proportion of algae (Figure 4A). The ZmCRD1 protein belonged to group III and was the closest to a homolog in *Sorghum bicolor*. Another MgPEC protein, ZmCRD2, was annotated in the protein database of NCBI. ZmCRD1 and ZmCRD2 shared 95.40% identity at the protein level and were located on the same branch (Figure 4A and Supplementary Figure 1). The p.T326 site of ZmCRD1 was highly conserved in Group III, particularly in monocotyledons and dicotyledons. The amino acid at the 326th position was alanine or serine in algae and was relatively random in Group I. The p.A44 site was not conserved in different species (Figure 4B). The amino acid sequences of ZmCRD1 in 37 maize inbred lines with *de novo* assembly were obtained from MaizeGDB, which represented a wide diversity of maize germplasms, including the 26 NAM founder lines. All 37 lines had only p.T326, while both p.A44 and p.T44 appeared (Figure 4C). All of these maize lines had normal green leaves. p.A44 and p.T326 were also conservative between ZmCRD1 and ZmCRD2 in B73 (Supplementary Figure 1). Taken together, p.T326M was more likely the causal mutation in *pgl*.

### ZmCRD1 and ZmCRD2 Function in Chloroplasts

**FIGURE 5:**
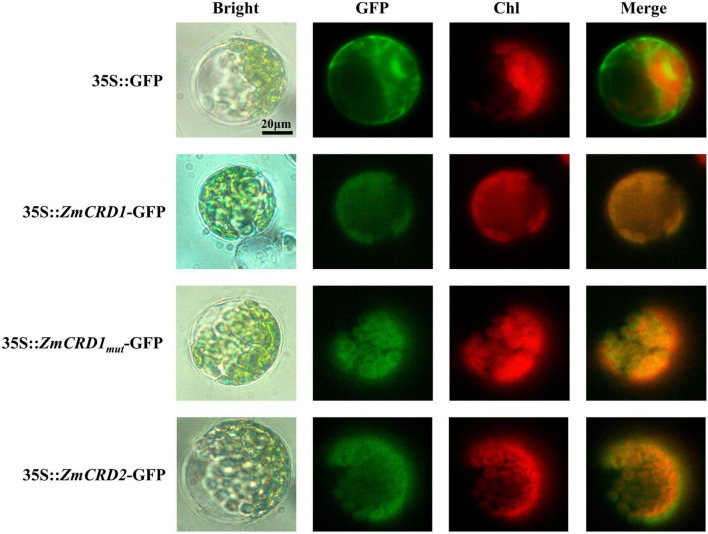
Subcellular localization of ZmCRD proteins in protoplasts of *Arabidopsis*. The GFP-tag was fused to the C-terminus of ZmCRDs. ZmCRD1_*mut*_ refers to ZmCRD1 with p.A44T and p.T326M mutations derived from the *pgl* mutant.

The phylogenetic analysis identified two CRD proteins, ZmCRD1 and ZmCRD2, that were subunits of MgPEC and highly similar in maize. To explore where ZmCRD proteins functioned in plant cells, we constructed four vectors for subcellular localization, including 35S::GFP, 35S::*ZmCRD1*-GFP, 35S::*ZmCRD1*_*mut*_ (ZmCRD1 with p.A44T and p.T326M mutations)-GFP and 35S::*ZmCRD2*-GFP, and transformed these vectors into *Arabidopsis* mesophyll protoplasts. The green fluorescence was spread over the whole protoplast for the GFP-only protein, while ZmCRD1-GFP, ZmCRD2-GFP and ZmCRD1_*mut*_-GFP fusion proteins only appeared in chloroplasts (Figure 5). In conclusion, ZmCRD1 and ZmCRD2 functioned in chloroplasts, and the p.A44T or p.T326M mutation did not alter the localization of ZmCRD1.

### Mutation of *ZmCRD1* Affects Chloroplast Morphology and Chlorophyll-Related Gene Expression

**FIGURE 6:**
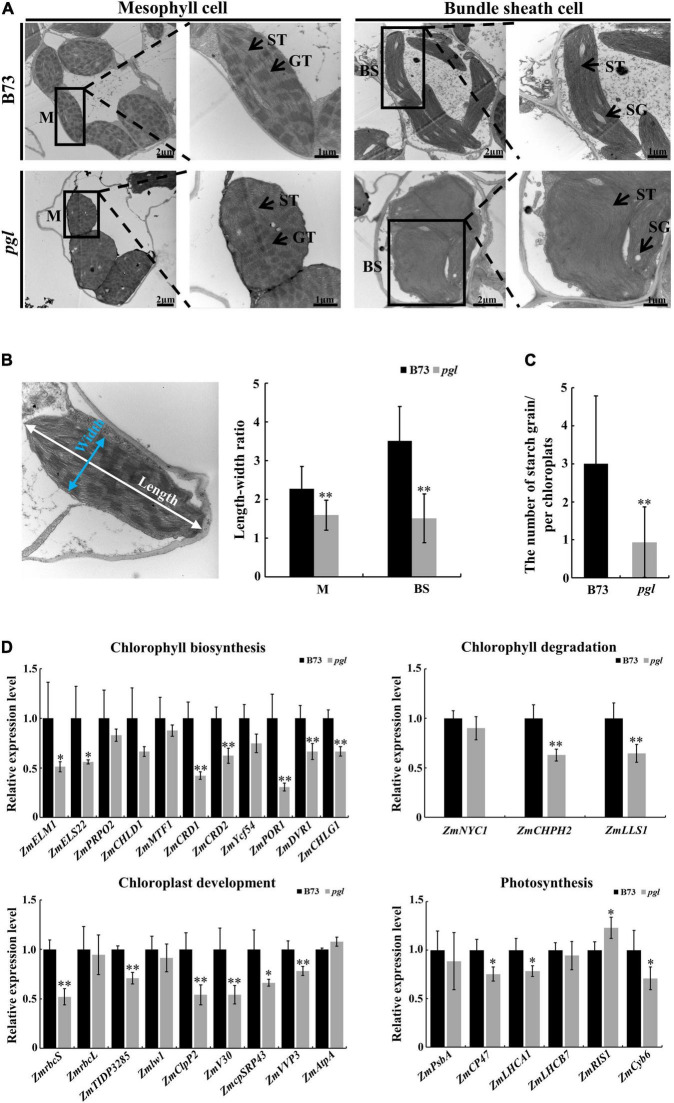
Ultrastructure observation of chloroplasts and expression pattern analysis between B73 and *pgl*. **(A)** The ultrastructure of chloroplasts in mesophyll cells and bundle sheath cells between B73 and *pgl*. M, mesophyll cell; BS, bundle sheath; ST, stroma thylakoid; GT, grana thylakoid; SG, starch grain. Three biological replications were performed. **(B)** The length-width ratio of chloroplasts was compared in mesophyll cells and bundle sheath cells between B73 and *pgl*. Data are presented as the means ± SD (*n* = 15). **(C)** The number of starch grains was counted in chloroplasts of bundle sheath cells between B73 and *pgl*. Data are presented as the means ± SD (*n* = 16). **(D)** qRT-PCR analysis of genes associated with chlorophyll biosynthesis, chlorophyll degradation, chloroplast development and photosynthesis. Data are presented as the means ± SD (*n* = 4). Asterisks represent significant differences between B73 and *pgl* detected by independent sample *T*-test (*P < 0.05, ^**^P < 0.01).

The ultrastructure of chloroplasts in B73 and *pgl* was observed at the third-leaf stage using transmission electron microscopy to identify whether chloroplast development was affected in the *pgl* mutant. The chloroplast structure of mesophyll cells was intact in *pgl* and stacked grana thylakoids did not change significantly compared to B73. However, the morphology of chloroplasts in mesophyll cells and bundle sheath cells changed in *pgl*. Chloroplasts of mesophyll cells tended to be spherical, while chloroplasts of bundle sheath cells showed an irregular morphology in *pgl* (Figure 6A). The length-width ratio of chloroplasts in *pgl* was significantly lower than that in B73 (Figure 6B). In addition, a few smaller starch grains were observed in the chloroplasts of bundle sheath cells in *pgl*, but oval and larger starch grains were observed in B73 (Figures 6A,C). These results indicated that *ZmCRD1* mutation affected chloroplast morphology and starch accumulation.

The *ZmCRD1* gene was expressed at higher level in leaves than in other organs, and its expression was lower in *pgl* compared to B73 (Supplementary Figure 2A). Moreover, we used a publicly RNA-seq data sets from 21 tissues spanning vegetative and reproductive stages of maize development (Walley et al., 2016). We found that the *ZmCRD1* gene had a higher expression level in the eighth mature leaf, internodes, silks and mature female spikelets (Supplementary Figure 2B). All these results indicated that *ZmCRD1* primarily functioned in photosynthetic tissues and organs.

Furthermore, we explored the expression pattern of protein-coding genes involved in chlorophyll biosynthesis, chlorophyll degradation, chloroplast development and photosynthesis (Figure 6B and Supplementary Table 8). All genes encoding enzymes involved in the chlorophyll biosynthesis pathway were down-regulated in *pgl* compared to B73, especially downstream genes of MgPEC, such as *protochlorophyllide oxidoreductase* (*ZmPOR1*), *divinyl protochlorophyllide reductase* (*ZmDVR1*) *and chlorophyllide a oxygenase* (*ZmCHLG1*). The expression levels of chlorophyll degradation related genes including *chlorophyllase* (*ZmCHPH2*) and *pheophorbide a oxygenase* (*ZmLLS1*) were significantly decreased in *pgl*. The expression of chloroplast development-associated genes that products function inside chloroplasts changed obviously. We found that *ribulose bisphosphate carboxylase small subunit* (*ZmrbcS*), *thylakoid lumen protein* (*ZmTIDP3285*), *ATP-dependent Clp protease proteolytic subunit* (*ZmV30*), *chloroplast protease complex* (*ZmClpP2*), *chloroplast signal recognition particle* (*ZmRP43*) and *vacuolar proton pump* (*ZmVVP3*) were down-regulated in *pgl* compared to B73. The transcriptional levels of light reaction related genes also showed various degrees of change. These results indicated that mutated *ZmCRD1* altered the expression of genes associated with chlorophyll biosynthesis and degradation, chloroplast development and photosynthesis in *pgl*.

### Mutation of *ZmCRD1* Leads to Abnormal Photosynthetic Capacity and Reduced Production

**FIGURE 7:**
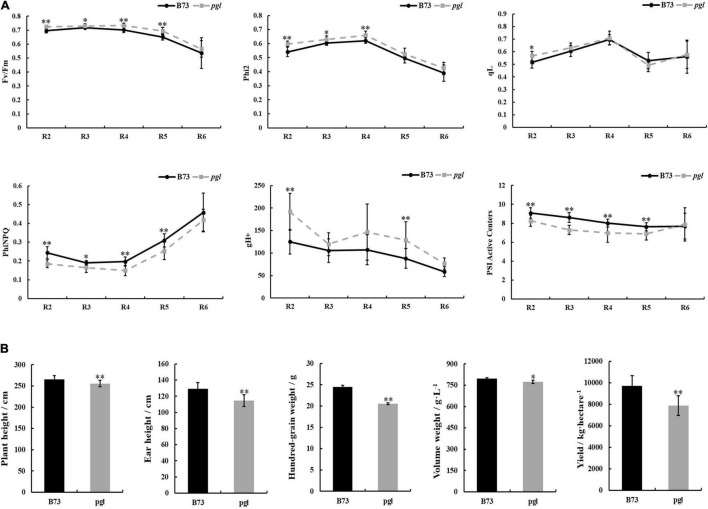
Chlorophyll fluorescence kinetic parameters of photosystem and comparison of production between B73 and *pgl* mutant. **(A)** The chlorophyll fluorescence parameters of the middle of ear leaves were measured in B73 and *pgl* at the reproductive stage. R2, blister stage; R3, milk stage; R4, dough stage; R5, dent stage; R6, physiological maturity stage. Data were calculated as the means ± SD (*n* = 8). **(B)** The agronomic traits were analyzed between B73 and *pgl*. Height data were calculated as the means ± SD (*n* = 10) and yield data were calculated as means ± SD (*n* = 3). Asterisks represent significant differences between B73 and *pgl* mutant detected by independent sample *T*-test (*P < 0.05, ^**^P < 0.01).

Light-harvesting is a primary function of chlorophyll molecules, and leaf color variation directly influences the photosynthetic capacity of photosystem II (PS II) and photosystem I (PS I) together with fluctuation in production. We measured chlorophyll fluorescence kinetic parameters, including the maximum quantum efficiency (*F*_*v*_/*F*_*m*_), actual quantum efficiency (Phi2), quantum yield of regulatory energy dissipation (PhiNPQ), quantum yield of photochemical quenching (qL), thylakoid proton conductivity (gH^+^) and PS I active centers, at different growth periods using the MultispeQ system (Figure 7A and Supplementary Figure 3). Phi2 and *F*_*v*_/*F*_*m*_ in *pgl* were higher than B73 suggesting that the *pgl* mutant had more effective actual light energy conversion efficiency and potential maximum light energy conversion efficiency. qL showed no obvious changes between B73 and *pgl*, which indicated that the photosynthetic activity of PS II might not be impaired in *pgl*. However, the PhiNPQ of *pgl* was decreased compared to B73, which indicated that *pgl* had a lower photoprotection capacity and was vulnerable to strong light. The gH^+^ was markedly increased meaning increased the activity of ATP synthase in the chloroplasts of *pgl*. The fraction of active PS I center that was operational to receive or pass electrons was less in *pgl*, especially at the reproductive stage. These results indicated that the photosynthesis was perturbed in *pgl*.

In addition, to study whether development and production of *pgl* were influenced, some important agronomic traits were analyzed at different developmental phases (Figure 7B). Plant height and ear height of *pgl* were lower than those of B73 at the pollen stage. Yield-associated traits, such as hundred-grain weight, volume weight and yield per hectare, were dramatically decreased in *pgl* compared to B73. Thus, *ZmCRD1* mutation led to developmental retardation and production reduction in maize.

## Discussion

### Functional Comparison of *ZmCRD1* and *ZmCRD2* in Maize

Chlorophyll biosynthesis is a complex biological process catalyzed by at least 17 different key enzymes, and a variety of cofactors and transcription factors participate in this process. Numerous chlorophyll-deficient maize mutants have been identified, such as l*-Blandy 4, *oro*, *oro2*, and *cf1* (Huang et al., 2009; Hung et al., 2021). Although the function of MgPEC in chlorophyll biosynthesis has been confirmed to catalyze the conversion of MgPME to DV Pchlide, its entire structure and components are not clear. The catalytic subunit CRD1 and regulatory subunit Ycf54 of MgPEC have been widely studied in photosynthetic organisms (Hollingshead et al., 2012, 2017; Yang et al., 2015; Herbst et al., 2018). In grass, *CRD1* gene has been cloned from four rice mutants, *m167*, *yl-1*, *ygl8*, and *ysl1*, and a barley mutant, *xantha-I*, exhibiting an obvious chlorotic phenotype (Rzeznicka et al., 2005; Sheng et al., 2017; Wang X. X. et al., 2017; Li et al., 2019). Our research first identified a MgPEC gene, *ZmCRD1*, involved in chlorophyll biosynthesis in maize via combining the BSA strategy with RNA-seq.

According to previous reports, only one *CRD* gene that mutation could cause chlorophyll-deficient phenotype has been identified in rice and *Arabidopsis* (Tottey et al., 2003; Bang et al., 2008; Wang X. X. et al., 2017; Li et al., 2019). Hexaploid wheat contains three sets of subgenomes (A, B, and D), consistently, there are three *CRD* genes. Of which, the most similar one to ZmCRD1 is located on chromosome 3D (Figure 4A). In maize, ZmCRD1 and ZmCRD2 were located on the same branch of the evolutionary tree with 95.40% identity (Figure 4 and Supplementary Figure 1) and targeted to chloroplasts (Figure 5). However, *ZmCRD2* expression decreased rather than increased in leaves of *pgl* (Supplementary Figure 2A). Moreover, we analyzed the expression pattern of these two genes using the publicly available RNA-seq data sets covering diverse stages and tissues in maize (Walley et al., 2016). *ZmCRD1* showed a very high abundance (FPKM from 0.1 to 692.9) in various tissues and organs, while *ZmCRD2* had a lower abundance (FPKM from 0.0 to 49.0) all the time (Supplementary Figure 2B) implying that *ZmCRD2* cannot complement the dysfunction of *ZmCRD1*, most likely because of its low expression in *pgl*. Therefore, *ZmCRD1* might play a more important role rather than *ZmCRD2* in chlorophyll biosynthesis. In addition, *ZmCRD1* and *ZmCRD2* were mainly expressed in the leaves, silks, internodes and mature female spikelets. *ZmCRD2* also had a higher abundance in the pericarp/aleurone and endosperm crown compared to *ZmCRD1*, which indicated that *ZmCRD1* and *ZmCRD2* might play same or specific roles in maize.

### The *ZmCRD1* Gene Functions in Chlorophyll Biosynthesis

**FIGURE 8:**
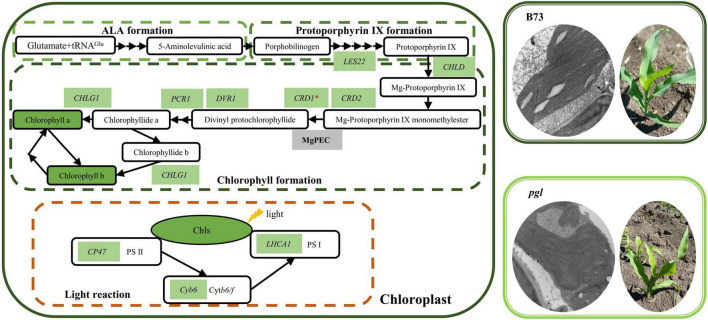
The model of chlorophyll biosynthesis and light reaction of photosynthesis in *pgl*. The chlorophyll biosynthesis pathway mainly consists of three parts (ALA formation, protoporphyrin IX formation and chlorophyll formation). Most genes and metabolites showed down-regulated expression in the *pgl* mutant compared to the wild-type plant (B73) resulting in decreased chlorophyll contents, abnormal chloroplast development and perturbed photosynthesis. The green background represents decreased expression of genes or metabolites in the *pgl* mutant. These genes are labeled on the basis of qRT-PCR analysis and are involved in chlorophyll biosynthesis and the light reaction of photosynthesis.

Based on our research and previously reported findings, we proposed a model of chlorophyll biosynthesis and light reaction of photosynthesis in *pgl* (Figure 8). CRD1 is considered to be a catalytic subunit of MgPEC. The *CRD1* mutation could decrease the activity of cyclase, accumulate more substrates (MgPME) while synthesize less products (Pchlide) in *chl27* and *xantha-I* (Tottey et al., 2003; Rzeznicka et al., 2005). In our study, the causal mutation of *ZmCRD1* was identified using positional mapping and phylogenetic analysis, and did not affect splicing of the *ZmCRD1* transcript, but changed amino acids of ZmCRD1 might affect cyclase binding to substrates or assembly of cyclase. As a consequence, the activity or stability of cyclase changes resulting in reduced chlorophyll contents.

In addition, *CRD1* mutation affected mRNA accumulation of chlorophyll biosynthesis, chloroplast development, and photosynthesis-related genes. In rice *crd1* mutants, *OsCRD1* is expressed at lower level because of a single nucleotide substitution. The upstream genes of *OsCRD1* are up-regulated, but the expression levels of downstream genes are decreased. Some key genes involved in chloroplast development and photosynthesis are also down-regulated expression (Sheng et al., 2017; Chen et al., 2018). Mutated *ZmCRD1* caused chlorophyll reduction together with down-regulated expression of other genes encoding enzymes involved in chlorophyll biosynthesis (Figure 6D). The lower expression levels or mutations of these genes also result in chlorophyll-deficient phenotypes. *PORB* encoding protochlorophyllide oxidoreductase is essential for maintaining light-dependent chlorophyll synthesis (Sakuraba et al., 2013). *DVR* gene encodes a divinyl reductase involved in chlorophyll synthesis of which mutant has yellow leaves (Wang et al., 2010, 2013). The expression of *CHLG* gene encoding chlorophyll synthase is limited leading to chlorophyll reduction and is involved in feedback-control of chlorophyll biosynthesis (Gaubier et al., 1995; Shalygo et al., 2009). Chloroplast development-associated genes were down-regulated expression and associated with abnormal chloroplast morphology in *pgl* (Figure 6D). For example, the chloroplast stroma-localized Clp protease belongs to ATP-dependent protease family and is essential for chloroplast development (Olinares et al., 2011; Xing et al., 2014). *CpSRP* genes encode chloroplast signal recognition particle proteins in plants that are involved in thylakoid biogenesis and their mutants show various chlorophyll-deficient phenotypes (Asakura et al., 2008; Kirst and Melis, 2014; Guan et al., 2016). The down-regulated expression of photosynthetic protein complex-related genes might affect the stabilization of photosystem and photosynthetic activity (Figure 6D). The CP47 protein is one of the core components of PS II and its deficiency may impair PS II activity (Putnam-Evans and Bricker, 1992; Alfonso et al., 1994). Lhca1 is a component of LHCI in PS I and forms a functional heterodimer with Lhca4 to harvest light in higher plants (Wientjes and Croce, 2011). Therefore, the mutation of *ZmCRD1* might affect activity of MgPEC resulting in a chlorophyll-deficient phenotype and could alter the gene expression pattern in multiple pathways inside chloroplasts.

### The Effects of *ZmCRD1* Mutation on Photosynthesis and Production

Chlorophyll molecules function to capture solar energy and are non-covalently associated with chlorophyll-binding proteins on the thylakoid membrane meaning that chlorophyll reduction may affect chloroplast development and photosynthetic capacity. Most chlorophyll-deficient mutants show lower photosynthetic efficiency and photosynthetic activity (Lv et al., 2015, 2020; Song et al., 2018). We found that although the light energy conversion efficiency of PS II was increased in *pgl*, the proportion of absorbed light energy used for regulative heat dissipation (PhiNPQ) was lower (Figure 7A), which meant that PS II of *pgl* easily suffers light-induced damage. The change of gH^+^ may be attributable to the activity of ATP synthase in chloroplasts and affects proton motive force, lumen acidification, electron transfer and the modulation of NPQ (Kanazawa and Kramer, 2002; Kanazawa et al., 2017). For the *cfq* mutant with altered ATP synthase regulation, the increased gH^+^ causes a series of changes in chloroplast, which may contribute to the accumulation of electrons on the acceptor side of PS I, and result in loss of PS I activity in *Arabidopsis* (Kanazawa and Kramer, 2002; Kanazawa et al., 2017). The gH^+^ value is also affected by light and the concentrations of CO_2_. Therefore, the chloroplast ATP synthase might play a role in regulating the activity of photosynthesis. In *pgl* mutant, the increased gH^+^ might also be accompanied by a change in the activity of chloroplast ATP synthase and regulate the activity of PS I consistent with decreased active PS I center (Figure 7A). The *CRD1* gene is required for maintaining the stability of PS I and light-harvesting complex I (LHCI) in *Chlamydomonas reinhardtii*, and the ratio of Cth1 (Copper target homolog 1) and CRD1, as di-iron enzymes, affects PS I and LHCI accumulation (Moseley et al., 2002, 2014). Therefore, mutated *ZmCRD1* gene prevented chlorophyll accumulation that might modulate the stabilization of chlorophyll-binding proteins. On the other hand, starch is a main photosynthetic product in photosynthetic organs. The amount, morphology and size of starch differ substantially in different species and environments. The morphology and number of starch grains were obviously different in the chloroplasts of *pgl* and B73 (Figures 6A,C). There were larger oval starch grains in bundle sheath cells of B73, while a few small round starch grains were observed in those of *pgl*. Therefore, the synthetic capability of photosynthetic products might be inhibited due to the mutation in *ZmCRD1*. As a consequence, the yield fell by 18.97% in *pgl* (Figure 7B).

## Data Availability Statement

The data presented in the study are deposited in the NCBI-SRA repository, and its accession number is PRJNA830983 (https://www.ncbi.nlm.nih.gov/bioproject/PRJNA830983).

## Author Contributions

YY, YX, SS, and HD planned and designed the research. YX, HD, HH, SL, XS, HL, HKL, and DX performed the experiments, analyzed the data and performed the field work. YX, HD, SS, and YY wrote the manuscript. All authors contributed to the article and approved the submitted version.

## Conflict of Interest

The authors declare that the research was conducted in the absence of any commercial or financial relationships that could be construed as a potential conflict of interest.

## Publisher’s Note

All claims expressed in this article are solely those of the authors and do not necessarily represent those of their affiliated organizations, or those of the publisher, the editors and the reviewers. Any product that may be evaluated in this article, or claim that may be made by its manufacturer, is not guaranteed or endorsed by the publisher.

## Footnotes

1
http://ftp.ebi.ac.uk/ensemblgenomes/pub/release-39/plants/fasta/zea_mays/

2
http://ccb.jhu.edu/software/hisat2/index.shtml

3
https://www.ncbi.nlm.nih.gov/protein/

4
https://www.ebi.ac.uk/Tools/msa/mview/

5
https://www.maizegdb.org/

6
https://qteller.maizegdb.org/
