# Supplementary material for: Mutation in Mg-Protoporphyrin IX Monomethyl Ester (Oxidative) Cyclase Gene ZmCRD1 Causes Chlorophyll-Deficiency in Maize
Source: Front Plant Sci. 2022 Jul 7;13:912215. doi: 10.3389/fpls.2022.912215 (PMC9301084; doi:10.3389/fpls.2022.912215)
Supplement: Supplementary file 1 [file Data_Sheet_1.docx]

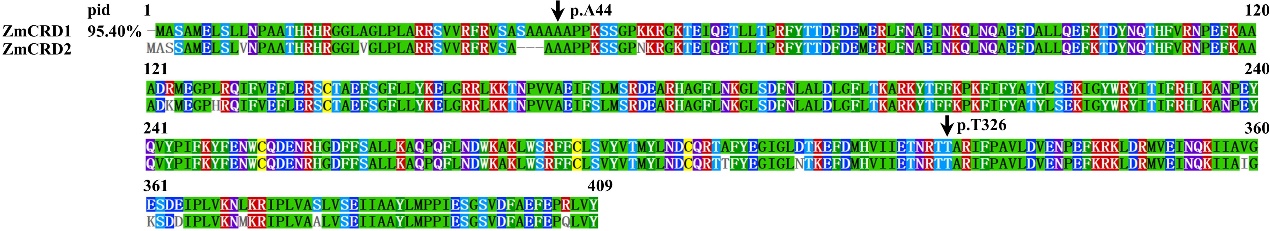


**FIGURE S1** The protein sequence alignment between ZmCRD1 and ZmCRD2 using Clustal Omega. The “pid” represents identity of proteins. The arrows point to p.A44 and p.T326 sites of ZmCRD1.


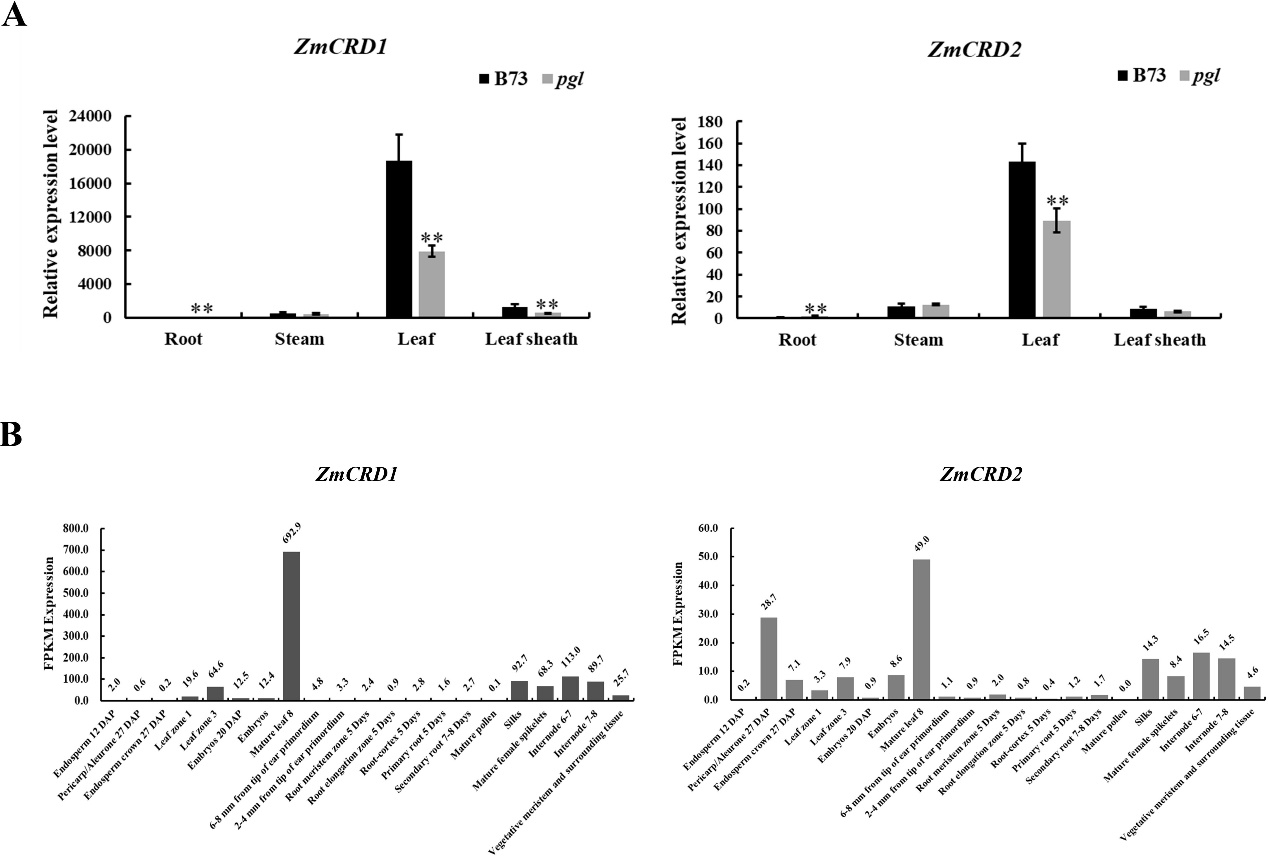


**FIGURE S2** The expression pattern analysis of *ZmCRD* genes. (A) The qRT-PCR analysis of *ZmCRD1* and *ZmCRD2* in different tissues and organs at the third leaf stage. Seedlings were grown to the third- leaf stage in greenhouse. Data are presented as the means±SD (n=5). Asterisks represent significant differences between B73 and *pgl* detected by Independent Sample T Test (*, P﹤0.05; **, P﹤0.01). (B) The expression pattern analysis (FPKM) of *ZmCRD1* and *ZmCRD2* in 21 maize tissues (Walley et al., 2016).


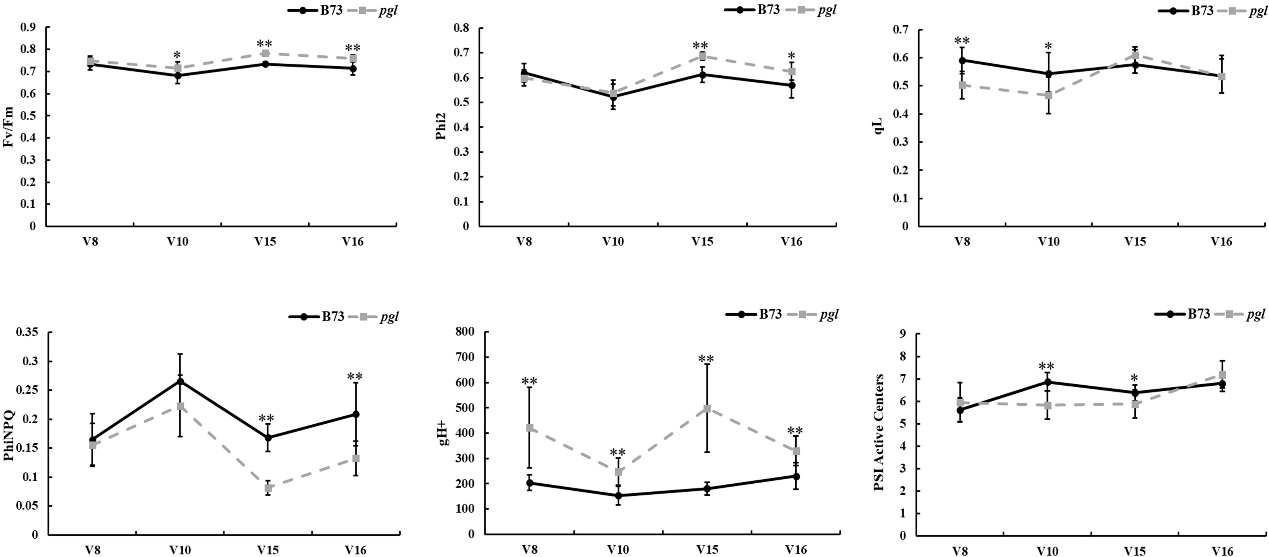


**FIGURE S3** The chlorophyll fluorescence parameters of the middle of canopy leaves were measured in B73 and *pgl* at the vegetative stage. Data are calculated as the means±SD (n=8). V8, eighth leaf stage; V10, tenth leaf stage; V15, fifteenth leaf stage; V16, sixteenth leaf stage. Asterisks represent significant differences between B73 and the *pgl* mutant detected by Independent Sample T Test (*, P﹤0.05; **, P﹤0.01).
