# Supplementary material for: Mutation in Mg-Protoporphyrin IX Monomethyl Ester (Oxidative) Cyclase Gene ZmCRD1 Causes Chlorophyll-Deficiency in Maize
Source: Front Plant Sci. 2022 Jul 7;13:912215. doi: 10.3389/fpls.2022.912215 (PMC9301084; doi:10.3389/fpls.2022.912215)
Supplement: Supplementary file 2 [file Data_Sheet_2.docx]

**Table S1** The mean values of parameters in the formulas used for calculating chlorophyll fluorescence kinetic parameters.

| **Parameter** | **Mean** | **Source** |
| --- | --- | --- |
| k | arbitrary correlation coefficient | (Maas and Dunlap, 1989; Raymond and Daughtry, 2014) |
| ref.Abs_650nm_ | intensity of emitted light at the 650nm (red light) wavelengths | (Maas and Dunlap, 1989; Raymond and Daughtry, 2014) |
| ref.Abs_940nm_ | intensity of emitted light at the 940nm (infrared light) wavelengths | (Maas and Dunlap, 1989; Raymond and Daughtry, 2014) |
| Abs_650nm_ | intensity of transmission light through the leaf at the 650nm wavelengths | (Maas and Dunlap, 1989; Raymond and Daughtry, 2014) |
| Abs_940nm_ | intensity of transmission light through the leaf at the 940nm wavelengths | (Maas and Dunlap, 1989; Raymond and Daughtry, 2014) |
| F_m_ | value of maximal fluorescence after dark adaptation | (Genty et al., 1989) |
| F_o_ | value of minimal fluorescence after dark adaptation | (Genty et al., 1989) |
| F’_m_ | value of maximal fluorescence in the light-adapted state | (Genty et al., 1989; Kramer et al., 2004; Kuhlgert et al., 2016) |
| F_s_ | relative steady-state fluorescence yield | (Genty et al., 1989; Kramer et al., 2004; Kuhlgert et al., 2016) |
| F’_o_ | value of minimal fluorescence in the light-adapted state | (Genty et al., 1989; Kramer et al., 2004; Kuhlgert et al., 2016) |
| τ | ECS decay time constant | (Kanazawa and Kramer, 2002) |
| P_M_ | maximal absorption difference between dark and the second saturation pulse taken after application of far-red light to oxidize electron carriers | (Kanazawa et al., 2017) |
| P_0_ | P_M_ value measured in dark adapted leaves prior to illumination with a full complement of active PSI centers | (Kanazawa et al., 2017) |

**Table S2** All primers used in this study.

| **Gene** | **Number of Primers** | **Forward primer (5’→3’)** | **Reverse primer (5’→3’)** |
| --- | --- | --- | --- |
| *ZmCRD1* | #1 | CCAGTCCATCGCCAACC | CCTTCGGCAGAAAGCACC |
|  | #2 | GGGGACAAGTTTGTACAAAAAAGCAGGCTTCATGGCCTCCGCCATGGA | GGGGACCACTTTGTACAAGAAAGCTGGGTTGTAAACGAGCCGGGGCTC |
| *ZmCRD2* | #3 | AGCTCGTCGCCAGTCGC | CGTGCTAATATGGACAATCATCTAC |
|  | #4 | GGGGACAAGTTTGTACAAAAAAGCAGGCTTCATGGCCTCCTCCGCCATGG | GGGGACCACTTTGTACAAGAAAGCTGGGTTGTAAACAAGCTGGGGCTC |
| *Zmcrd1* | #5 | CTAGAGTCTGAACGAATGAACCA | CGAAAGCGGACCACGGA |
|  | #6 | CTAGAGTCTGAACGAATGAACCA | GAAGCCAACGCCATCGCCTCCATTTCGTCGAAT |

**Table S3** Genetic segregation ratios of different F_1_ offspring and the F_2_ populations.

| **Cross combination** | **Phenotype of F_1_** | **F_2_ population** | | **Ratio** | **χ^2^** | **p-value** |
| --- | --- | --- | --- | --- | --- | --- |
|  |  | **Normal green** | **pale green** |  |  |  |
| B73×*pgl* | green | 2854 | 885 | 3.22 | 0.11 | 0.74 |
| Mo17×*pgl* | green | 165 | 53 | 3.11 | 0.05 | 0.82 |
| Chang7-2×*pgl* | green | 200 | 69 | 2.90 | 0.06 | 0.80 |
| P125×*pgl* | green | 218 | 67 | 3.25 | 0.34 | 0.56 |
| 4cv×*pgl* | green | 177 | 60 | 2.95 | 0.01 | 0.91 |

χ^2^﹤χ^2^_0.05_=3.84, p﹥0.05

**Table S4** Statistics data of RNA-seq in two libraries.

| **Libraries** | **Clean reads** | **Correct recognition rate of bases (%)** | **Mapped ratio (%)** | **The number of genic SNPs** | **The number of intergenic SNPs** |
| --- | --- | --- | --- | --- | --- |
| WP | 28,889,383 | 94.51 | 96.21 | 48,541 | 9,309 |
| MP | 25,211,877 | 94.80 | 96.50 | 44,211 | 7,034 |

**Table S5** The primer sequences of polymorphic SNP markers used for the genotype using HRM method.

| **Polymorphic SNP markers** | **Position** | **Ref** | **Alt** | **Forward primer (5’→3’)** | **Reverse primer (5’→3’)** |
| --- | --- | --- | --- | --- | --- |
| snp3 | 560222 | T | C | CTTGTCAGCCAGGAGCCG | CGTTGTCTTTGCCATAAGCT |
| snp7 | 1239205 | G | A | GTGTTGGGGCCAGCATT | GAGGTTCTTTGGCTTACTTTGT |
| snp10 | 1465500 | G | A | AAGGAAGGGGACGACGGG | GGCAATGTTCTTTGACCACC |
| snp15 | 1591068 | C | T | ATTGCACTCAGCTTAGGGG | ATGATGATACAACTGCGATTACTT |
| snp20 | 1889112 | A | G | TTTTATCTACGTCGGGCTGTT | GCATGGTAGAAAGATTCGTCA |
| snp24 | 1983989 | A | G | CAGACCCAGCAAGAACAACC | GGACGGAAGAGGAGCACAG |
| snp26 | 2035745 | C | A | CCCCTAGTGAAGAACCTGAAG | CGAAATCAACCGAGCCAGAC |
| snp29 | 2202962 | A | G | TGCTGAATTTCAGGATCATAGC | TACACTTTCTCCAGTCCAACG |
| snp31 | 2258462 | C | T | TGCTAATAAAGAAGCCCTTGC | CTCCCCTTTCGTCCACAA |
| snp33 | 2453649 | A | C | ATGATGACCTCCTCGTATTGAA | ATCATGGATGCCTTTCGTT |
| snp34 | 2490820 | T | C | TGTTTGTGCAGAAGAAATCCA | TGAGGACGGTCTTGTAGCC |
| snp39 | 2564760 | T | C | TGCATGGTTTGTCAGGGTC | TTAAAGCAGATTATGTTGTGGGT |
| snp43 | 2673166 | T | C | TCTGATTATAGGGACGGTGTTAG | AATACCTCAATGATTTGTTGCA |
| snp48 | 3989600 | A | G | TGAGCTTTGATCTTTAACTGATATG | CAGGCAAAGGCAGCACTA |
| snp52 | 4784721 | A | G | GTCTGGTTGATGGATACGGAA | AGCTGGCAAGATTAAAATGC |
| snp61 | 2070478 | C | T | CTGATTGCAGCGTACCAGAA | GCATTAGTACATGGAAAATTAAGAG |

**Table S6** The phenotypic identification and genetic analysis of *pgl* and *Zmcrd1*.

| **Genotype** | **The number of green plants** | **The number of yellow plants** | **ratio** | **p-value** |
| --- | --- | --- | --- | --- |
| A_2_a_2_×A_1_A_1_ | 56 | 0 | - | - |
| A_2_a_2_×A_1_a_1_ | 43 | 13 | 3.31:1 | 0.758 |
| A_2_a_2_×a_1_a_1_ | 25 | 31 | 1:1.24 | 0.423 |
| A_2_A_2_×A_1_A_1_ | 56 | 0 | - | - |
| A_2_A_2_×A_1_a_1_ | 56 | 0 | - | - |
| A_2_A_2_×a_1_a_1_ | 56 | 0 | - | - |

**Table S7** The annotated information of 18 CRD proteins in different species used for the phylogenetic analysis.

| **NCBI accession** | **Gene annotation** | **Species** | **Taxonomy (Family)** |
| --- | --- | --- | --- |
| PKA48142.1 | Magnesium-protoporphyrin IX monomethyl ester [oxidative] cyclase, chloroplastic | *Apostasia shenzhenica* | Orchidaceae |
| XP_020585474.1 | Magnesium-protoporphyrin IX monomethyl ester [oxidative] cyclase, chloroplastic | *Phalaenopsis equestris* | Orchidaceae |
| XP_020701805.1 | Magnesium-protoporphyrin IX monomethyl ester [oxidative] cyclase, chloroplastic | *Dendrobium catenatum* | Orchidaceae |
| AUY61990.1 | Magnesium-protoporphyrin IX monomethyl ester [oxidative] cyclase | *Cymbidium sinense* | Orchidaceae |
| AOS51483.1 | Magnesium-protoporphyrin IX monomethyl ester [oxidative] cyclase | *Calamus jenkinsianus* | Palmae |
| XP_008778346.2 | Magnesium-protoporphyrin IX monomethyl ester [oxidative] cyclase, chloroplastic | *Phoenix dactylifera* | Palmae |
| XP_010910140.1 | Magnesium-protoporphyrin IX monomethyl ester [oxidative] cyclase, chloroplastic isoform X1 | *Elaeis guineensis* | Palmae |
| XP_020108573.1 | Magnesium-protoporphyrin IX monomethyl ester [oxidative] cyclase, chloroplastic | *Ananas comosus* | Bromeliaceae |
| XP_003567276.1 | Magnesium-protoporphyrin IX monomethyl ester [oxidative] cyclase, chloroplastic | *Brachypodium distachyon* | Gramineae |
| XP_044975891.1 | Magnesium-protoporphyrin IX monomethyl ester [oxidative] cyclase, chloroplastic | *Hordeum vulgare* | Gramineae |
| XP_044348576.1 | Magnesium-protoporphyrin IX monomethyl ester [oxidative] cyclase, chloroplastic | *Triticum aestivum* | Gramineae |
| XP_006644062.2 | Magnesium-protoporphyrin IX monomethyl ester [oxidative] cyclase, chloroplastic | *Oryza brachyantha* | Gramineae |
| XP_015622109.1 | Magnesium-protoporphyrin IX monomethyl ester [oxidative] cyclase, chloroplastic | *Oryza sativa* | Gramineae |
| AKU36802.1 | Magnesium-protoporphyrin IX monomethyl ester cyclase | *Phyllostachys edulis* | Gramineae |
| RLM91293.1 | Magnesium-protoporphyrin IX monomethyl ester cyclase | *Panicum miliaceum* | Gramineae |
| XP_004967679.1 | Magnesium-protoporphyrin IX monomethyl ester [oxidative] cyclase, chloroplastic | *Setaria italica* | Gramineae |
| NP_001309212.1 (ZmCRD1) | Magnesium-protoporphyrin IX monomethyl ester [oxidative] cyclase, chloroplastic | *Zea mays* | Gramineae |
| XP_020405783.1 (ZmCRD2) | Magnesium-protoporphyrin IX monomethyl ester [oxidative] cyclase, chloroplastic | *Zea mays* | Gramineae |
| XP_002457655.1 | Magnesium-protoporphyrin IX monomethyl ester [oxidative] cyclase, chloroplastic | *Sorghum bicolor* | Gramineae |

**Table S8** The primer sequences used for qRT-PCR analysis

| **Gene ID** | **Gene Name** | **Forward primer (5’→3’)** | **Reverse primer (5’→3’)** |
| --- | --- | --- | --- |
| Zm00001d008230 | *ZmCRD1* | AGCCCCGGCTCGTTTAC | GGTTTTCACCATTCATCCAA |
| Zm00001d040463 | *ZmCRD2* | GAGCCCCAGCTTGTTTACTG | TCCTTTGACCATCCCAGTTT |
| Zm00001d029027 | *ZmYcf54* | ACGCAGAATCAGTTGAAGAGG | ATTGCTGAACTTTGGAGGC |
| Zm00001d011876 | *ZmELM1* | ATAAAAGCAAATGAGGATAACACC | GGCAGCAAAATCAAGAACC |
| Zm00001d029074 | *ZmLES22* | AACTTGCCTCTGATACTCTACGC | CCAATCTTTTCCTGCCCTCT |
| Zm00001d003214 | *ZmPRPO2* | CGTAGAGGGGCAACCAAC | TTATTTCCTGCGTAGAAGAACC |
| Zm00001d013013 | *ZmCHLD1* | TTAGCTCATGGCCTAAGTACAG | ATTAGCTCTTCCATCGGTGATT |
| Zm00001d018411 | *ZmMTF1* | AAGGCTTTCTTCGGTTCATT | TCATTTCACTCTGCTTCACATTA |
| Zm00001d001820 | *ZmPOR1* | CAAGAAGCTCTGGGAGATCAG | GGTACACATAATACAACGAAGCG |
| Zm00001d029150 | *ZmDVR1* | TTCCGGGAGAAAGAAACTTGTA | GAGTCGTGTTTACATCATCAGC |
| Zm00001d037984 | *ZmCHLG1* | CAGAAAACGAGGTAATAACCCA | CACCCACAGCAAGATAAAACA |
| Zm00001d039312 | *ZmNYC1* | TTCACCTGGTATGGTCCTCA | CTTTCCACTTCCTTTTACAACTC |
| Zm00001d032926 | *ZmCHPH2* | AGTGTTCTTGTCCTACTACCTGCTG | GGGTGAGGCTGACGCTATG |
| Zm00001d027656 | *ZmLLS1* | CACAAGGTTACTGGACGAAGA | TAACAAGGGGCCTCAAAAG |
| Zm00001d052595 | *ZmrbcS* | GCGAGTACATACATACTAGGCA | CTCGAACTTCTTGTTGCCGTAG |
| V00171.1 | *ZmrbcL* | AACTTTCCAAGGTCCGCCAC | TGATTTCACCAGTTTCGGCTTG |
| Zm00001d033896 | *Zmlw1* | GCATCTGGCTCACCAACGAA | GCTCCAAATGCAGGCAACAC |
| Zm00001d028895 | *ZmCLPP2* | ACCCGAACCCCAACACTA | CCACCTGATCGACCAAATC |
| Zm00001d047634 | *ZmV30* | CCAGAGCGTCGTCAGTCAG | CGTCGAGGTAGAGCAGTTGT |
| Zm00001d027518 | *ZmcpSRP43* | CTCCGAACCTGAACCCTCC | CGTCATCACCTCCCTTGCTT |
| Zm00001d031484 | *ZmTIDP3285* | GCGACGACAGTCTGCCACT | CGATGCCGAGCAATAAGG |
| Zm00001d053765 | *ZmVPP3* | ACCTTACCACTGGGTTCCG | GGCAGCCATTATTGTCTTACA |
| GRMZM5G875287 | *ZmATPa* | CGGATGAATTAGTCGTTGTTG | AAGAGCTTGAATACCGCTTGTA |
| Zm00001d000417 | *ZmPsbA* | CTGTAGTTGATAGCCAAGGTCG | TGAGCATTACGTTCGTGCA |
| GRMZM5G808939 | *ZmCP47* | TATGGTGGCGAACTTAATGG | CCTTGGACTGCTGCGAAA |
| Zm00001d006663 | *ZmLHCA1* | GTCAGGTTGATGGCCGATATAT | GAGCATTCCATCATTGACAGTC |
| Zm00001d039040 | *ZmLHCB7* | GGTTTTCGGTTTGTACCATGAT | GATAAAATCGGCAGGAACGAAA |
| Zm00001d016134 | *ZmRIS1* | CGCTGAGAACAAGTTCATCTG | AGGCATACATAGGTCTCATGTG |
| GRMZM5G800780 | *ZmCyb6* | GTCAGCAAGTATGATGGTTCTAAT | ACGCCTGTGACCCAAGTT |
